# Supplementary material for: Structures revealing mechanisms of resistance and collateral sensitivity of Plasmodium falciparum to proteasome inhibitors
Source: Nat Commun. 2023 Dec 14;14:8302. doi: 10.1038/s41467-023-44077-2 (PMC10721928; doi:10.1038/s41467-023-44077-2)
Supplement: Supplementary file 1 — Supplementary information [file 41467_2023_44077_MOESM1_ESM.pdf]

Structures revealing mechanisms of resistance and collateral sensitivity of *Plasmodium falciparum* to proteasome inhibitors

Hao-Chi Hsu,[1] Daqiang Li,[2] Wenhu Zhan,[2] Jianxiang Ye,[2] Yi Jing Liu,[3] Annie Leung,[3] Junling Qin,[4] Benigno Crespo,[5] Francisco-Javier Gamo,[5] Hao Zhang,[2] Liwang Cui,[4,6] Alison Roth,[7] Laura A Kirkman,[2,3] Huilin Li,[1*] and Gang Lin[2*]

[1] Department of Structural Biology, Van Andel Institute, 333 Bostwick Ave NE, Grand Rapids, MI 49503, United States

[2] Department of Microbiology & Immunology, Weill Cornell Medicine, 1300 York Avenue, New York, NY 10065, United States

[3] Division of Infectious Diseases, Department of Medicine, Weill Cornell Medicine, 1300 York Avenue, NY, 10065, United States

[4] Department of Internal Medicine, Morsani College of Medicine, University of South Florida, Tampa, FL 33612, United States

[5] Global Health Medicines R&D, GlaxoSmithKline, Severo Ochoa 2, 28760, Tres Cantos, Madrid, Spain

[6] Center for Global Health and Infectious Diseases Research, College of Public Health, University of South Florida, Tampa, Florida, United States

[7] Department of Drug Discovery, Experimental Therapeutics Branch, The Walter Reed Army Institute of Research, 503 Robert Grant Ave., Silver Spring MD, 20910

*Corresponding to H.L (huilin.li@vai.org) or G.L. (gal2005@med.cornell.edu)

Supplementary Information for

Structures revealing mechanisms of resistance and collateral sensitivity of Plasmodium falciparum to proteasome inhibitors

By Hsu et al.

This document contains 1 Supplemental Table and 8 Supplemental Figures

Supplemental Table 1. Cryo-EM data collection, 3D reconstruction and refinement

	**Pf20S-TDI-8304**	**c-20S-TDI-8304**	**Pf20Sβ6^{A117D}-WLW-vs**
**PDB (EMDB) code**	8G6E (EMD-29764)	8UD9 (EMD-42148)	8G6F (EMD-29765)
**Data collection and processing**			
Magnification	105,000x	105,000x	105,000x
Voltage (kV)	300	300	300
Electron dose (e^-/Å)	66	58	60
Dose per frame (e^-/Å)	0.88	1.16	0.92
Defocus range (μm)	-1.3 to -1.8	-1.0 to -1.4	-1.3 to -1.8
Pixel size (Å)	0.828	0.828	0.828
Symmetry imposed	C2	C2	C2
Initial number of particles	657,006	2,072,882	337,322
Final number of particles	305,581	1,250,898	74,883
Map resolution (Å)	2.18	2.04	2.58
Map resolution range (Å)	2.17-3.38	1.83-3.51	1.79-7.22
FSC threshold	0.143	0.143	0.143
**Refinement**			
Initial model used	6MUW	5LF3	8G6E
Model resolution (Å)	2.3	2.1	2.7
FSC threshold	0.5	0.5	0.5
Map-sharpening B-factor (Å^2)	-51.3	-69.5	-78.1
Model composition			
Non-hydrogen atoms	52,266	49,955	52,394
Protein residues	6,490	6,264	6,466
Ligands	6	2	4
Water	218	1120	599
B-factors (Å^2)			
Protein	21.44	26.07	43.79
Ligand	19.22	24.61	39.01
Water	15.56	25.69	36.35
r.m.s. deviations			
Bond lengths (Å)	0.004	0.004	0.004
Bond angles (°)	0.570	0.552	0.508
**Validation**			
MolProbity score	1.10	1.00	1.05
Clashscore	3.00	2.23	2.66
Poor rotamers (%)	0.56	0.15	0.37
Ramachandran statistics (%)			
Favored	98.01	98.28	98.18
Allowed	1.99	1.72	1.82
Outliers	0	0	0

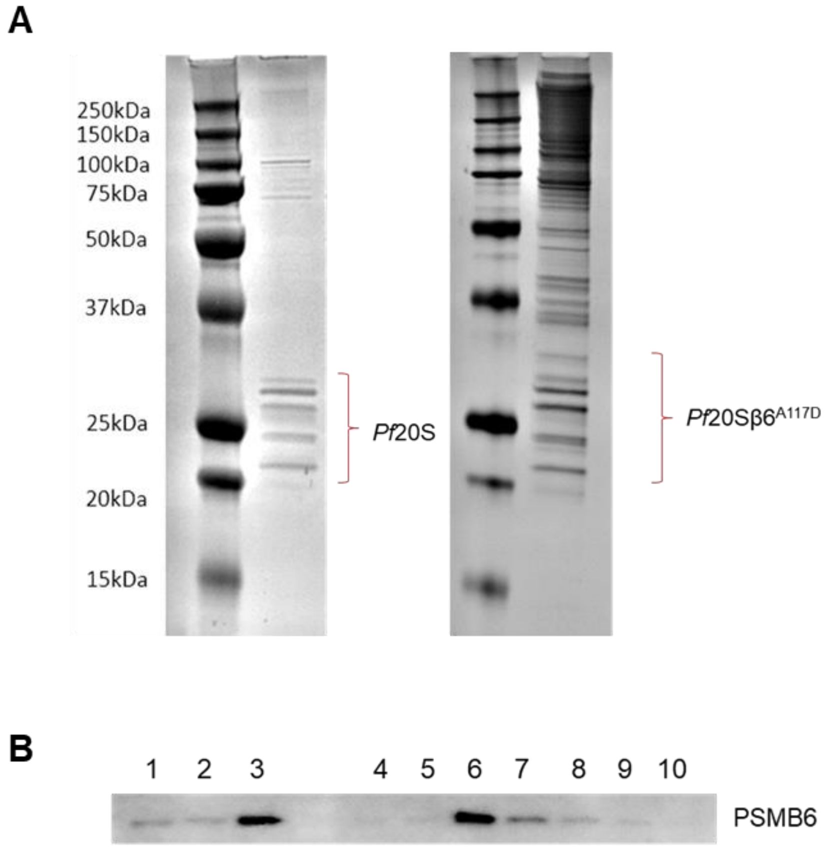

1. Pf20S β6A117D, 1,000 ng
2. Pf20S-WT, 1,000 ng
3. Hu c-20S, 50 ng
4. Pf20S β6A117D, 500 ng
5. Pf20S-WT, 500 ng
6. Hu c-20S, 50 ng
7. Hu c-20S, 20 ng
8. Hu c-20S, 10 ng
9. Hu c-20S, 5 ng
10. Hu c-20S, 2 ng

Impurity of Pf20S: 2.7 % Hu c-20S
Impurity of Pf20S β6^{A117D}: 3.2% Hu c-20S

Supplementary Figure 1. **Characterization of the Pf20S preparations used in the structural study.** **a)** SDS-PAGE gels of highly enriched wildtype Pf20S from *P. falciparum* NF54 (left) and the mutant Pf20Sβ6^{A117D} from Dd2 cells (right). **b**) Western blot estimation of the amount of the contaminating human constitutive proteasome (hu c-20S) copurified with the malaria proteasomes. Anti-PSMB6 (β1) antibody (Cell Signaling Technology, Cat. No. 13267) was used for the Western Blot. Hu c-20S (R&D, E-360) was used for titration and determination of the c-20S content in the enriched Pf20S samples. ImageJ was used for density quantification.

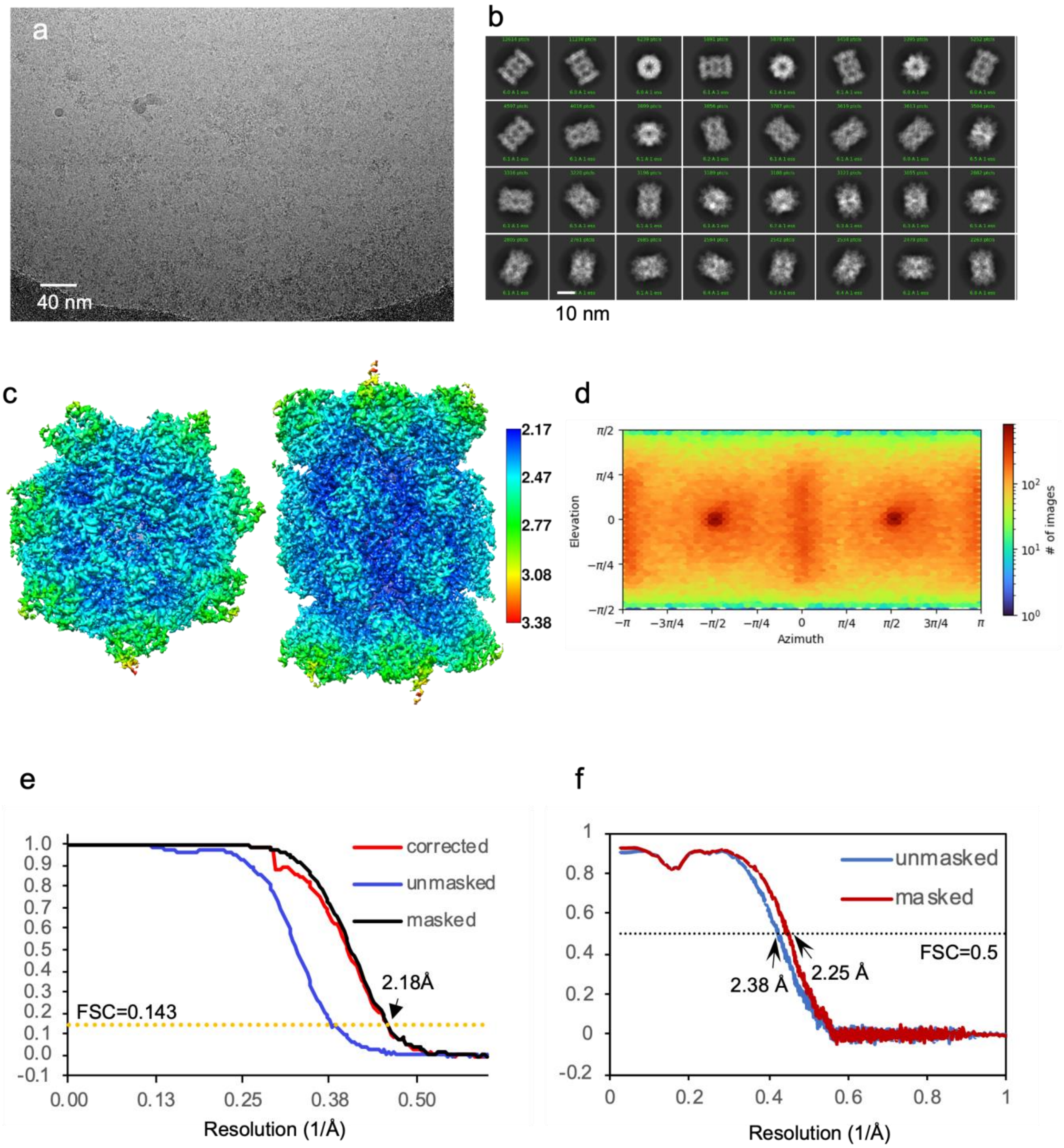

Supplementary Figure 2. **Cryo-EM 3D map of Pf20S-TDI-8304. a**) A typical raw micrograph after motion correction. **b**) Selected 2D class averages. **c**) Top and side views of EM map surface rendered and then colored coded by the local resolution estimation. **d**) The angular distribution of particles used in the final reconstruction. **e**) Fourier shell correlation plots. The average resolution was 2.18 Å at the

correlation threshold of 0.143. **f**) The calculated model-map FSC curves using the Phenix Mtriage program.

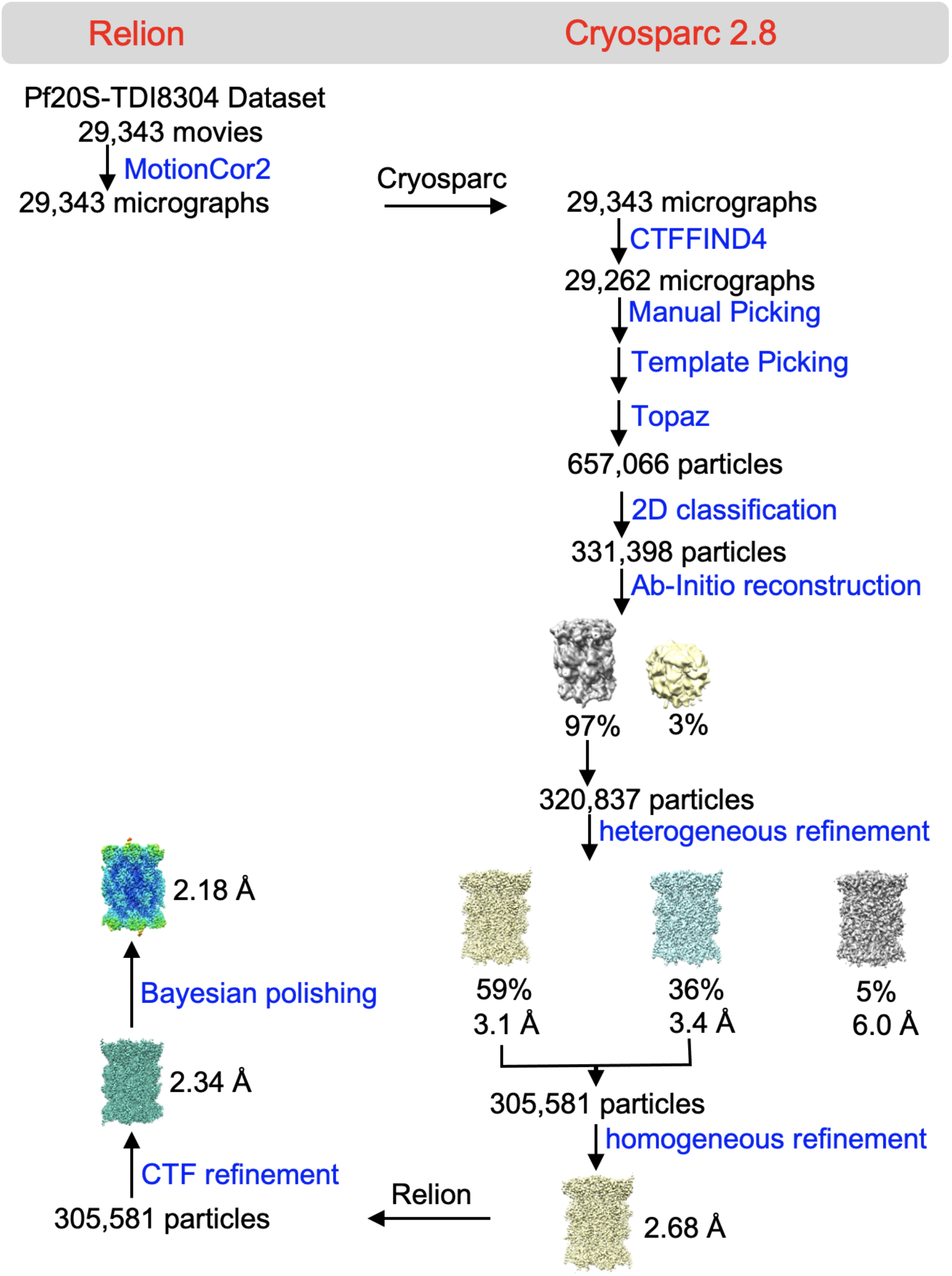

Supplementary Figure 3. **Workflow for image processing and 3D reconstruction of the TDI-8304 bound Pf20S complex structure.**

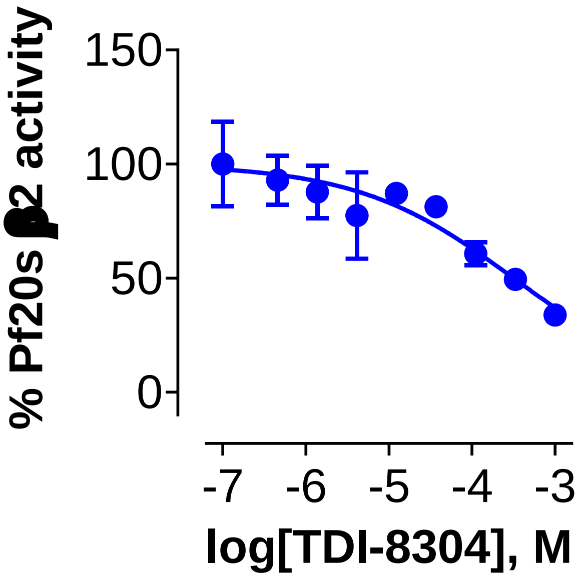

Supplemental Figure 4. Inhibition profile of the Pf20S β2 activity by 8304. A representative experiment is shown from three independent experiment. Mean ± SD**.**

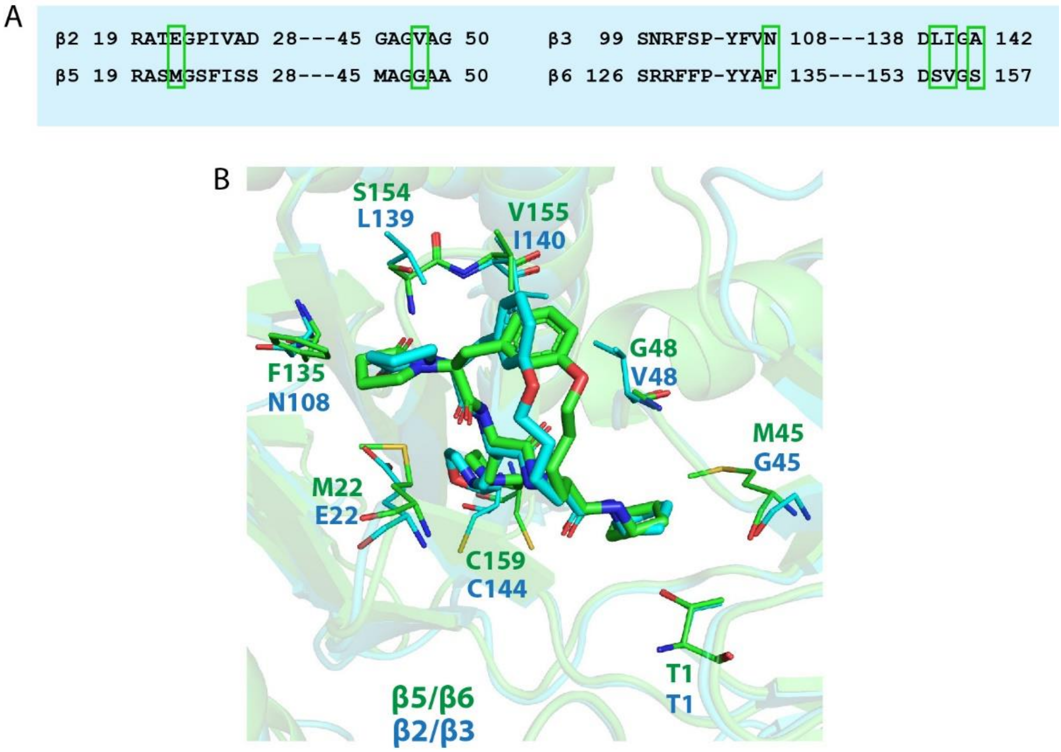

Supplemental Figure 5. Comparison of the Pf20S catalytic pockets. a) Amino acid sequence alignment at regions that line the substrate binding pockets of β2 and β5. **b**) Superimposition of substrate pockets of β5 and β2 both bound to TDI-8304.

a

```
Hsβ1 70  LNEP-----------------------------------------------------  73
Pfβ1 70  NRKKGRFHEGETIYDETTYDEEIDIDSINYLDYNNNNDNNLVTKNKYFYEDKFNDYN 126

Hsβ7 115 YADGES----------------------------------- 120
Pfβ7 118 INSQKYDNNDDNVLLYTNKNNDDEQNEYKNNEEYKEIHKDDL 159
```

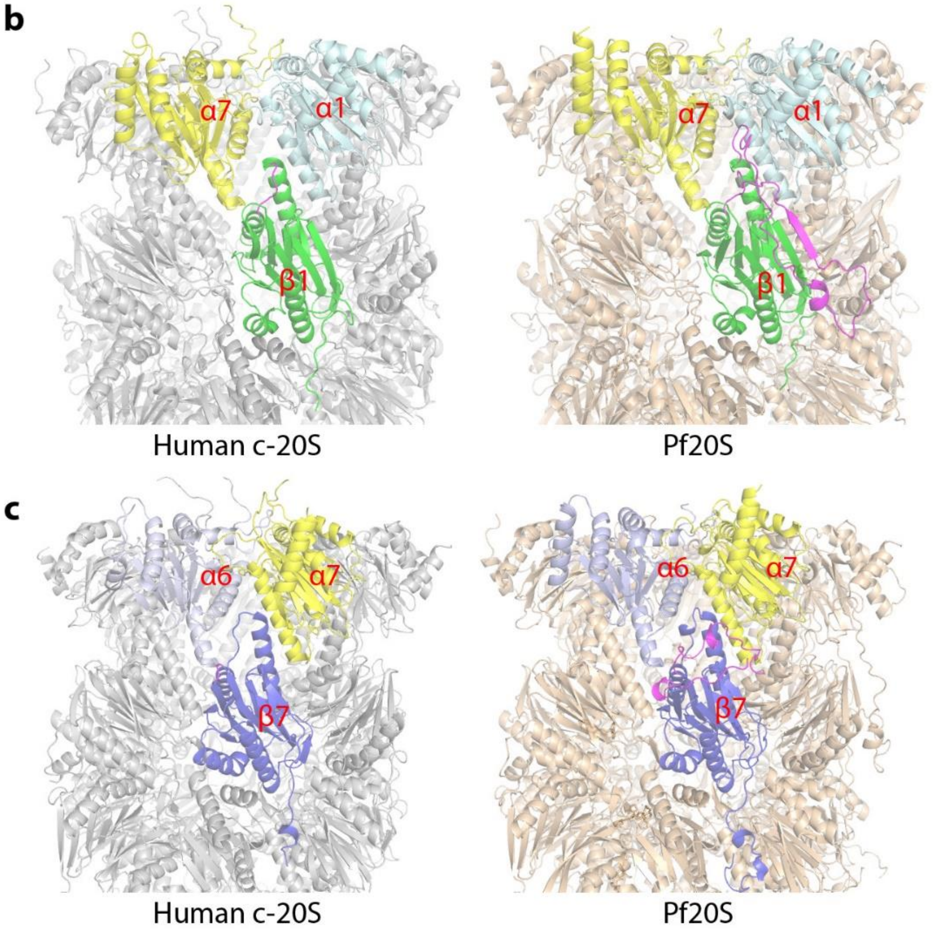

Supplemental Figure 6. Pf20S proteasome has two more loops in the β1 and β7 than the human proteasome. **a**) Structure-based sequence alignment showing that the two long loops in Pf20S are rich in asparagine (in dark green) and acidic amino acids (in red). **b**) The 4-residue loop between Leu70 and Pro73 of human β1 (left, magenta) is expanded to a 57-residue long loop (Asn70 to Asn126) in Pf20S β1 (right, magenta). **c**) The 5-residue loop between Tyr115 and Ser120 of human β7 (left, magenta) is expanded to a 42-residue long loop composed of Ile118 to Leu159 in the Pf20S β7 (right, magenta).

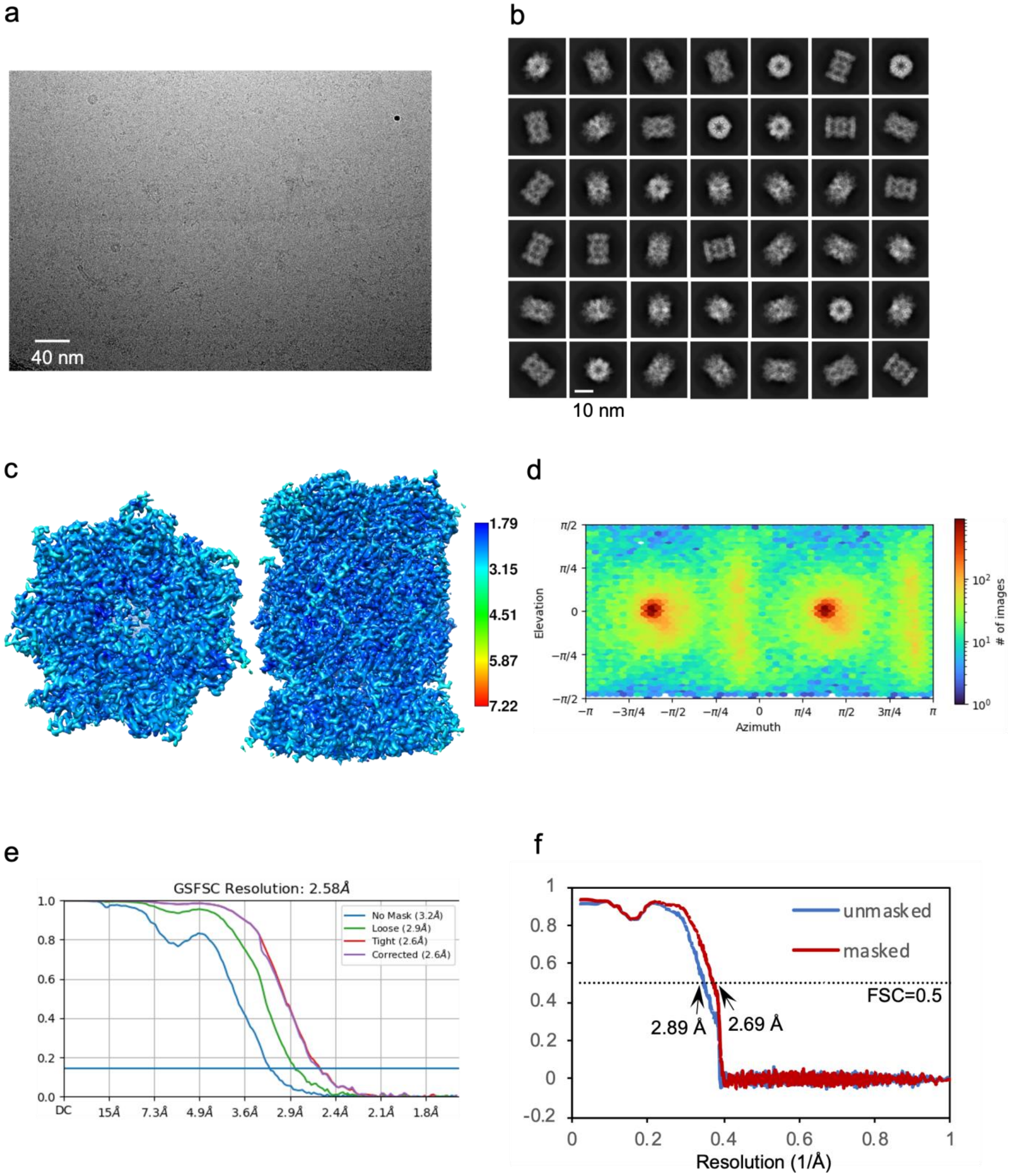

Supplemental Figure 7. Cryo-EM map of Pf20Sβ6^{A117D}-WLW-vs. **a**) Micrographs after motion correction. **b**) 2D averages. **c**) Top and side views of EM map colored coded by the estimated local resolutions. **d**) The angular distribution of particles used in the final reconstruction. **e**) Fourier correlation plot. The resolution was determined at FSC=0.143. **f**) The calculated model-map FSC curves by Mtriage in the Phenix.

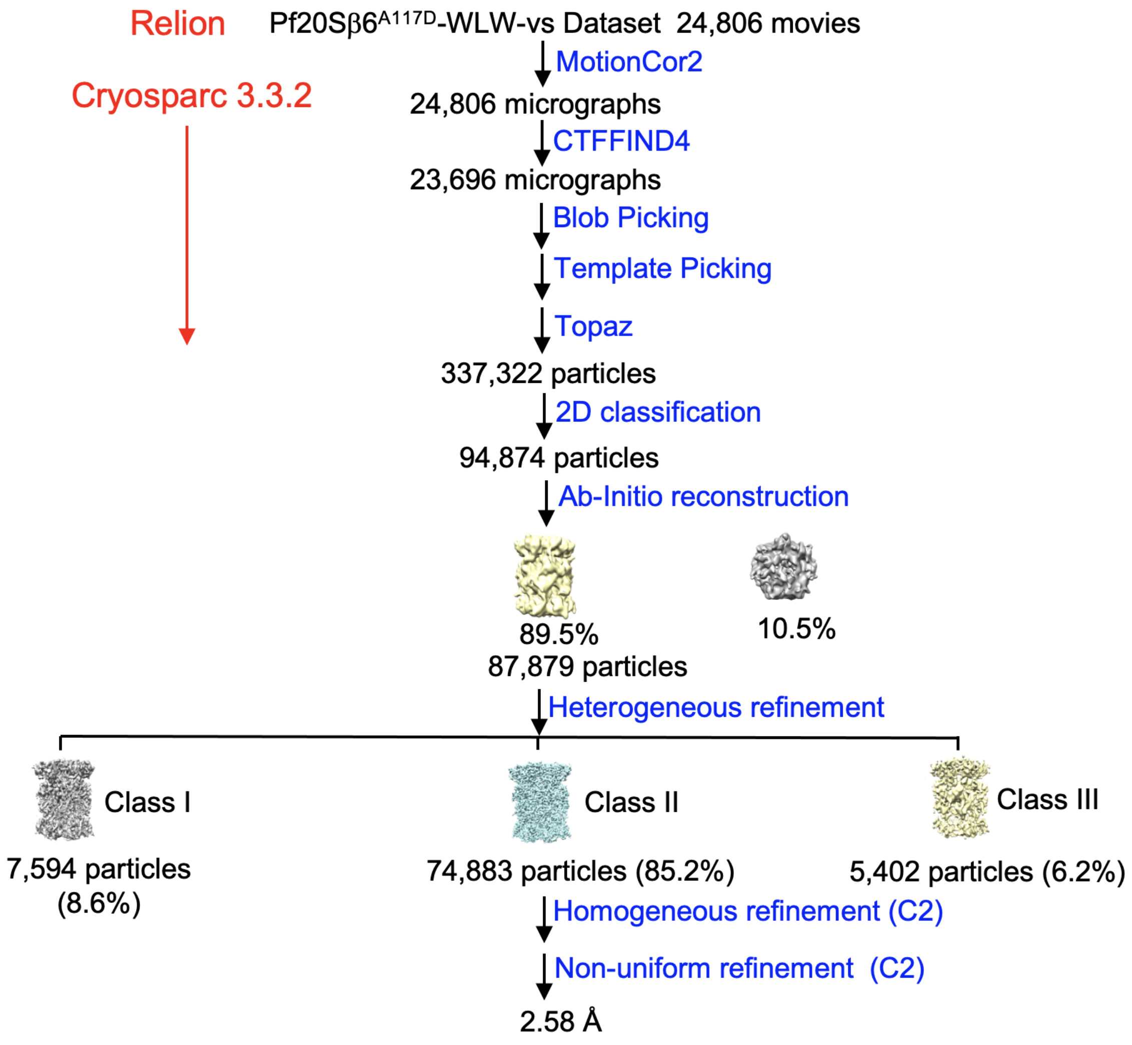

Supplemental Figure 8. The flow-chart for processing Pf20Sβ6^{A117D} with WLW-vs.

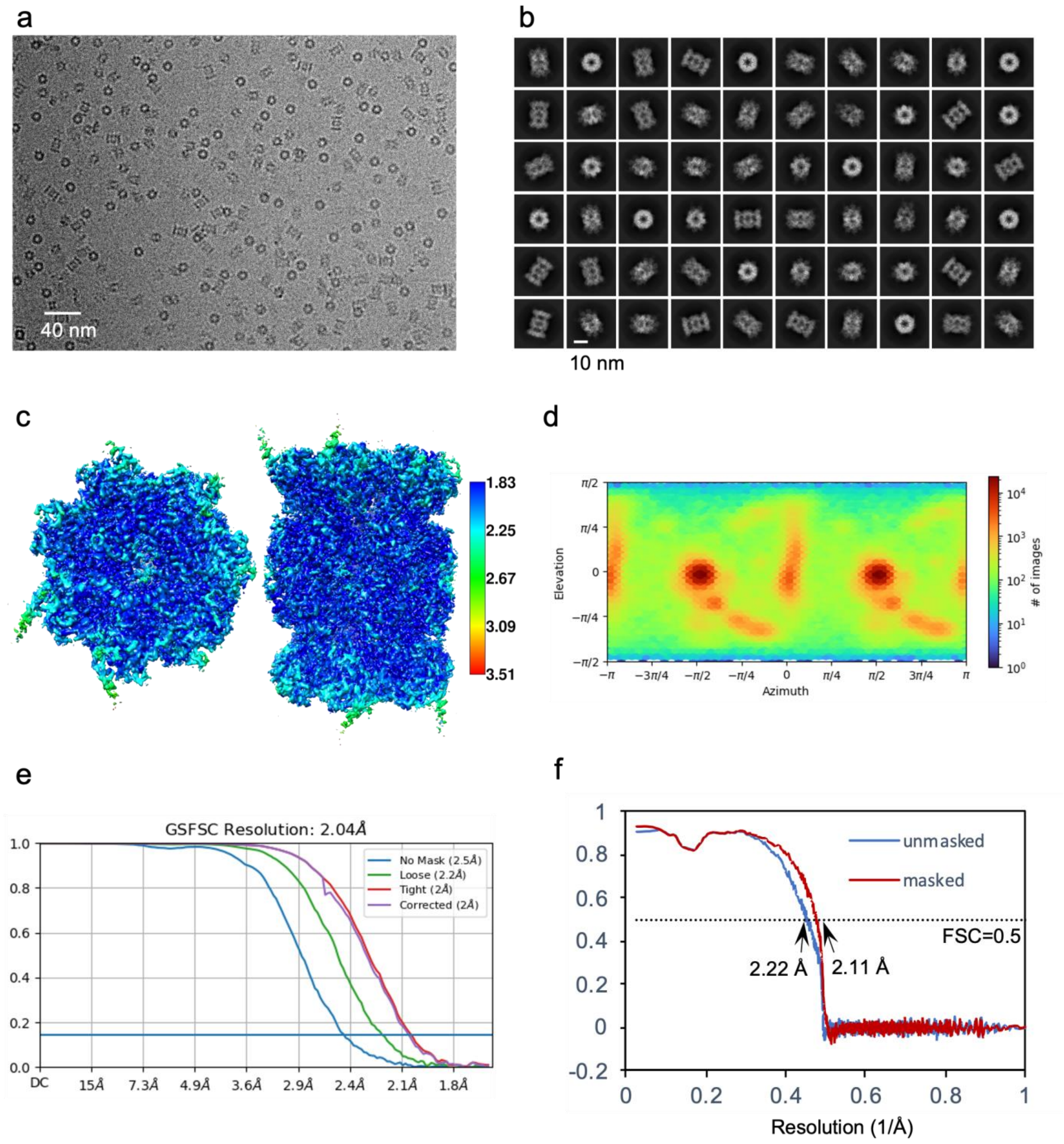

Supplemental Figure 9. Cryo-EM map of the human 20S-TDI-8304 complex. **a**) Micrographs after motion correction. **b**) 2D averages. **c**) Top and side views of the EM map color coded by the estimated local resolutions. **d**) The angular distribution of particles used in the final reconstruction. **e**) Fourier correlation plot. The resolution was determined at FSC=0.143. **f**) The calculated model-map FSC curves by Mtriage in the Phenix.

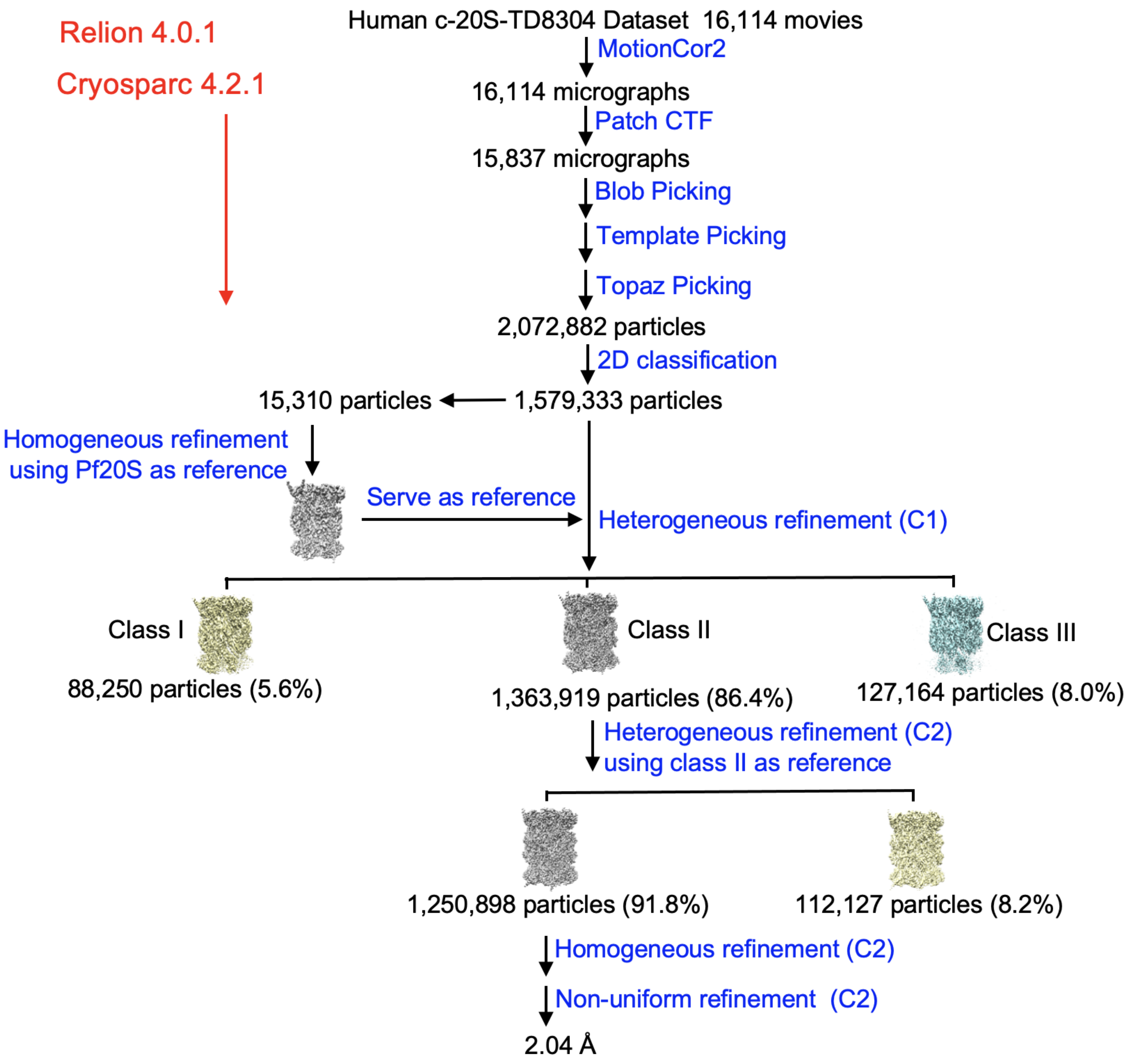

Supplemental Figure 10. The flow-chart for processing the cryo-EM images of the human c-20S-TDI-8304 complex.
